# Childbirth fear and its associated factors among pregnant women attending antenatal care at Gondar city public health institutions, northwest Ethiopia, 2022

**DOI:** 10.1371/journal.pone.0328819

**Published:** 2025-07-23

**Authors:** Wubedle Zelalem Temesgan, Mastewal Belayneh Aklil, Tazeb Alemu Anteneh, Selam Yibeltal Desalegn

**Affiliations:** 1 Department of Clinical Midwifery, School of Midwifery, College of Medicine and Health Sciences, University of Gondar, Gondar, Ethiopia; 2 Department of midwifery, school of nursing and midwifery, College of Medicine and Health Sciences, Wollo University, Dessie, Ethiopia; Haramaya University, ETHIOPIA

## Abstract

**Background:**

Childbirth is a normal physiological process that transit women to motherhood, and it is a major event in women’s life. Women face many challenges from conception to the childbirth process and during the postpartum period. Childbirth fear is one of the problems women encounter during pregnancy and it is associated with a wide range of negative outcomes on the health of the woman and her fetus. Even though childbirth fear has had negative health outcomes studies on the prevalence and its associated factors are scares in developing countries including Ethiopia.

**Methods:**

An institutional based cross-sectional study was conducted among 453 pregnant women from February 15–28/2022. Data were collected using a pretested, interviewer administered questionnaire, and a systematic sampling technique was employed to select the study participants. Epi Data version 4.6 and SPSS 25 were used for data entry, cleaning and, analysis, respectively. A binary logistic regression (bi- and multivariable) model was fitted to identify factors associated with childbirth fear. The level of significant association was declared using the adjusted odds ratio (AOR) with its 95% confidence interval (CI) and a p-value of ≤ 0.05.

**Results:**

Childbirth fear among pregnant women was 23.8% (95% CI: 20.1–28.0). Being rural residence (AOR = 6.24, 95% CI: 3.05, 12.80), having moderate social support (AOR = 0.54, 95% CI: 0.29, 0.99), anxiety during pregnancy (AOR = 2.82, 95% CI: 1.52, 5.23), and intimate partner violence (AOR = 4.95, 95% CI: 2.78, 8.81), were significantly associated with childbirth fear.

**Conclusion:**

The study showed that the magnitude of childbirth fear is high in the study area. Policymakers should develop strategies or screening tools for the early identification of women with pregnancy-related anxiety and childbirth fear, and it is important to give special attention and counseling to pregnant women in rural areas and victims of intimate partner violence.

## Introduction

Childbirth is a normal physiological process that transit women to motherhood and it is a major life event [1]. Women face many challenges from conception to the childbirth process and during the postpartum period. Childbirth fear is one of the problems women encounter during pregnancy and can be a source of distress for the women, their family, and their caregivers [2]. It is described as feelings of uncertainty and anxiousness before, during, or after delivery by thinking about the future labor and delivery or experiencing others’ fearful responses to childbirth and labor pain. Childbirth fear ranges from minor worth and anxieties about giving birth to severe fear of childbirth that has a considerable impact on women’s life, causing distress and affecting their mental well-being [3,4].

The prevalence of childbirth fear increases from time to time. According to a systemic review and meta-analysis report in 2022, FOC affects 16% of women globally [5]. Evidence showed that the prevalence of severe childbirth fear in Africa ranges from 8%−20% [6,7]. According to studies conducted in Arba Minch, Ethiopia, and Wollega zone, Ethiopia, 24.5% and 28.9% of pregnant women are affected by childbirth fear respectively [8,9].

Childbirth fear has negative outcomes on the health of the women and their fetuses such as prolonging the time to subsequent delivery, prolonging the active phase of labor, increasing the risk of operative delivery, use of epidural anesthesia, increasing the presence of post-traumatic stress syndrome and postpartum depression, and reduces mother to child bonding after delivery. It also has bad obstetric outcome including abortion, still birth, uterine rupture and preterm labor, [4,10,11]. The women’s fear is related to an inability to cope with labor pain, intervention during labor, fear of harm to self in labor and postnatal, fear of harm or stress to the baby, fear of not having a voice in decision making, fears about their body’s ability to give birth and fear of being abounded and alone [3]. Even though there is no definitive treatment for childbirth fear, studies suggested that cognitive behavioral therapy, hypnotherapy, antenatal education, psychoeducation, enhancing midwifery care, and intervention during labor are effective in reducing childbirth fear [4,12]. evidences showed that being nulliparous, having unplanned pregnancies, not having living children, pregnancy-related anxiety, depression, and perceived low levels of health related quality of life are some of the predictive factors for childbirth fear [6,8,13,14].

Nowadays, maternal and neonatal mortality reduction is a global priority. One of the Sustainable Development Goal (SDG) targets is to reduce the global maternal mortality ratio (MMR) to less than 70 maternal deaths per 100, 000 live births and neonatal mortality rate to at least 12 per 1,000 live births by 2030 [15]. Similarly, the Ethiopian government planned to reduce the MMR from 401 to 279 maternal deaths per 100,000 live births by 2024/25 [16], and doing a lot to decrease maternal and neonatal morbidity and mortality. But a little concern is given on the psychological aspects of pregnancy and childbirth. Even though childbirth fear is a common problem with multiple consequences, little is known in low-income countries including Ethiopia. Therefore, this study aimed to assess the childbirth fear and its associated factors among pregnant women attending ANC at Gondar city public health facilities in northwest Ethiopia. It could also provide input for stakeholders to design strategies for improving maternal and neonatal health.

## Methods and materials

### Study design, period, and setting

An institutional based cross-sectional study was conducted from February 15–28, 2022 in Gondar city public health institutions.

The city is found in Amhara regional state, Central Gondar Zone. It is located 166 km from Bahir Dar, the capital city of Amhara regional state, and 750 km northwest of Addis Ababa (the capital city of Ethiopia). According to the Population projection of Ethiopia for all regions at the woreda level from 2014–2017, the total population of the city was estimated to be 306,246. Among these 156,276 were females [17].

There are one governmental specialized hospital, eight governmental health centers, twenty-two health posts, one private primary hospital, and one general private hospital serving the community. All public health institutions in the city are providing ANC services.

### Study population and eligibility criteria

The study population was all pregnant women who had ANC follow-up at public health institutions in Gondar city, and Women who were seriously ill during the data collection period were excluded from the study.

### Sample size determination and sampling procedure

The single population proportion formula was used to calculate the sample size for this study by considering the following assumptions: the proportion of childbirth fear among pregnant women as 24.5% [8], level of confidence-95%, and margin of error-4%.

Therefore,


$$The\;sample\;size\;(n)\;=\frac{\left(Z\alpha /2{)}^{2*}p(1-p\right)}{d^{2}}\;\frac{=\;(1.96{)}^{2*}0.245(1-0.245)}{(0.04)2}=444\mathrm{\backslash ]}$$

After considering a non-response rate of 5%, we obtained a total sample size of 466.

**Fig 1:**
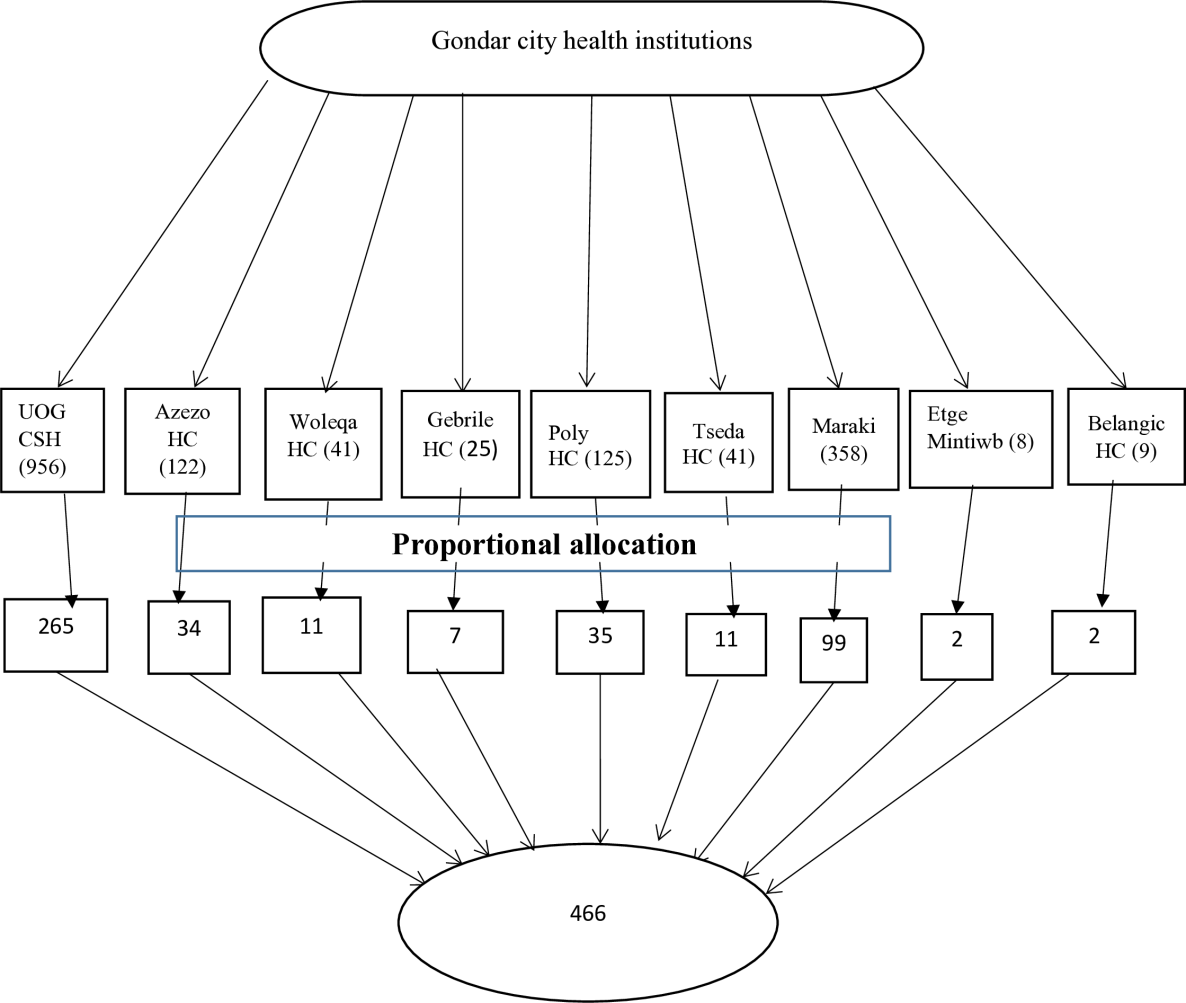
Schematic presentation of the sampling procedure of childbirth fear and its associated factors among pregnant women attending antenatal care at Gondar city public health institutions, northwest Ethiopia, 2022.

In Gondar city, there is one specialized referral hospital and eight public health centers. First, all public health facilities in Gondar city were considered. Then, the calculated sample size was proportionally allocated to each health facility based on the number of pregnant women who visited health facilities during the preceding two weeks before data collection. The sampling interval was calculated for each health institution, which was 3. The first case in each facility was selected randomly using a lottery method. Finally, a systematic random sampling technique was used to select all eligible pregnant women (Fig 1).

### Variables of the study

**Dependent variable**: Childbirth fear.

**Independent variables**: women’s age, residence, religion, women educational status, women occupation, marital status, husband’s educational status, husband’s occupation, age at first marriage, gravidity, gestational age, planned current pregnancy, wanted current pregnancy, mode of delivery of the preceding child, previous pregnancy complication, previous adverse pregnancy outcome, the preferred mode of delivery, preconception care, history of mental illness, history of chronic illness, social support, anxiety during pregnancy, depression during pregnancy, intimate partner violence and perceived health-related quality of life (HRQoL) were the independent variables.

### Operational definitions and measurement

**Childbirth fear:** Wijma Delivery Expectation/Experience Questionnaire (W-DEQ) Version A was used to measure childbirth fear. This 33-item rating scale has a 6-point Likert scale as a response format, ranging from ‘ not at all’ (= 0) to ‘ extremely’ (= 5) yielding a score range between 0 and 165, pregnant women who scored ≥ 85 were considered as having childbirth fear [8,18])

**Antenatal Depression**: The Edinburgh Postnatal Depression Scale (EPDS) was used to detect depression. The EPDS is a 10 item questionnaire, scored from 0 up to 3 (a higher score indicating more depressive symptoms), pregnant women who scored 12 and above were categorized as depressed women while pregnant women who scored below 12 were considered as none depressed [19].

**Anxiety during pregnancy:** was measured using a revised version of the 10-item pregnancy-related anxiety Questionnaire (PRA-Q). Each item was on a 4-point Likert scale of 0–3 and Participants who scored above 13 were considered as having pregnancy-related anxiety [20].

**Social support:** is defined as the physical and psychological comfort provided by other people. It was measured using the Oslo three-item social support scale. The respondents who scored 3–8 on the Oslo three-item social support scale were categorized as having poor support, 9–11 were categorized as having moderate support, and 12–14 were categorized as having strong support [21].

**Intimate partner violence:** An intimate partner is considered as a current spouse, co-habited, current boyfriend, former partner, or spouse. If the respondent said “Yes” to any one of the ranges of sexual, psychological, physical, or any combination of the three coercive acts regardless of the legal status of the relationship with the current/former intimate partner, it was considered as intimate partner violence [22].

**Health-related quality of life:** was measured using short version of WHO quality of life (WHOQOL-BREF) questionnaire. It contains a total of 26 items from which 24 items are categorized into four domains (physical, psychological, social relationships, and environmental). The remaining two questions were scored individually to assess the perception of a person about their quality of life and overall health. The respondents who scored above the mean for the transformed score were considered as having high HRQoL [23].

**Preconception care utilization:** If women get at least one type of intervention, either treatment or advice and lifestyle modification care at least once before pregnancy to 3 months after pregnancy [24].

### Data collection tool and procedures

The data collection tool was developed by reviewing related literature [18,21,22,25]. Data were collected using semi-structured, pretested questionnaires through face-to-face interview. The questionnaire contains socio-demographic characteristics, obstetric-related variables, intimate partner violence-related questions, and questions assessing social support, antenatal depression, anxiety during pregnancy, health related quality of life, and childbirth fear. Nine BSc midwives and one MSc clinical midwife were recruited for data collection and supervision respectively.

### Data quality control measures

The data collection tool was prepared

in English and then translated into the local language, Amharic, and then translated back to English to maintain consistency. Before the actual data collection, the questionnaire was pretested on 5% of the total sample size at Koladiba Primary Hospital. One-day training was provided for data collectors and supervisor about the aim of the study, contents of the tool, sampling technique, and also how to give clarification and adequate description for the participants. During data collection, data collectors were supervised for any difficulties. The consistency and completeness of the data were checked by the data collectors and supervisor and the incomplete data were discarded before data entry.

### Data processing and analysis

Data were checked, coded, and entered into Epi Data version 4.6 and then exported to SPSS version 25 for analysis**.** Bivariable logistic regression was done to identify variables candidates for multivariable logistic regression, and variables having a p-value less than 0.2 were entered into multivariable logistic regression analysis. Adjusted odds ratio with 95% CI and a p-value of ≤ 0.05 was used to determine the level of significance. The variance inflation factor (VIF) was checked for multicollinearity and was acceptable with a value of <10, in our analysis the maximum value was 1.806. Moreover, the Hosmer Lemeshow goodness of fit was done to check the model fit and it was 0.085.

### Ethical consideration

Ethical clearance was obtained from the University of Gondar ethical review committee (VP/RTT/05/336/2021). Support letters were submitted to the health facilities, and permission for facilities was obtained from administrates. Written informed consent was taken from each study participant after a clear explanation of the aim of the study. Study participants were also informed that they had the full right to withdraw from the interview at any time.

## Results

### Socio-demographic characteristics of study participants

In this study, a total of 453 pregnant women were involved with a response rate of 97.21%. The age of the participants ranges from 20 to 40 years with a mean age of 27.2(± 4.23 SD) years. Most of the study participants (85.7%) were urban residents. The majority of the respondents (91.6%) and (98%) were orthodox Christian in religion and married in their marital status respectively (Table 1).

**Table 1:** Socio-demographic characteristics of pregnant women who were attending antenatal care in Gondar city public health facilities, northwest Ethiopia, 2022 (n = 453).

Variables	Frequency	Percent
**Maternal age**		
≤24 years	130	28.7
25–29 years	178	39.3
30–34 years	101	22.3
≥35years	44	9.7
**Residence**		
Urban	388	85.7
Rural	65	14.3
**Religion**		
Orthodox Christian	415	91.6
Muslim	32	7.1
Others^*^	6	1.3
**Maternal educational status**		
No formal education	89	19.6
Primary education	48	10.6
Secondary education	164	36.2
College and above	152	33.6
**Maternal Occupational status**		
Government Employed	120	26.5
Housewife	178	39.3
Self-employed	90	19.9
Student	39	8.6
Others^**^	26	5.7
**Husband education status**		
No formal education	80	18.0
Primary education	46	10.4
Secondary education	97	21.8
College and above	221	49.8
**Husband Occupational status**		
Farmer	51	11.5
Private employee	164	36.9
Government employee	181	40.8
Merchant	42	9.5
Others^***^	6	1.4

Others^*^; Protestant and Jewish, Others^**^; Merchant & daily laborer, Unmarried^*^; Single &Widowed, Others^***^; Student & Daily laborer.

### Obstetrics-related characteristics of study participants

Of the study participants, 242 (53.4%) were multigravida. Regarding the gestational age, more than half (58.1%) were greater than 28 weeks of gestation. More than three-fourths (85.4%) of study participants had planned pregnancy. The majority (89.9%) of the study participants preferred vaginal delivery and more than one-fourth (28.7%) of women’s had preconception care. The Majority (98.7%) of the respondents’ current pregnancy is wanted and about 95.4% and 96.2% of study participants had no history of mental and chronic illness respectively (Table 2).

**Table 2:** Obstetrics-related characteristics of pregnant women who were attending antenatal care in Gondar city public health facilities, northwest Ethiopia, 2022 (n = 453).

Variables	Frequency	Percent
**Age at first marriage**		
<18 years	53	11.8
18–24 years	299	66.6
≥ 25 years	97	21.6
**Gravidity**		
Primigravida	211	46.6
Multigravida	242	53.4
**Current gestational age**		
≤28 weeks	190	41.9
>28 weeks	263	58.1
**Planned current pregnancy**		
Yes	387	85.4
No	66	14.6
**Mode of delivery of the preceding pregnancy**		
Vaginal delivery	213	89.9
Cesarean delivery	24	10.1
**Previous pregnancy complication**		
Yes	44	18.2
No	198	81.8
**Previous Adverse pregnancy outcome**		
Yes	50	20.7
No	192	79.3
**Preferred mode of delivery**		
Vaginal delivery	429	94.7
Cesarean delivery	24	5.3
**Had preconception care**		
Yes	130	28.7
No	323	71.3

### Psychosocial related variables

More than one-third (35.3%) of the study participants experience anxiety during pregnancy. Also, nearly one-fourth (24.3%) of women faced intimate partner violence during this pregnancy (Table 3).

**Table 3:** Psychosocial-related characteristics of study participants in Gondar city, Northwest Ethiopia, 2022 (n = 453).

Variables	Frequency	Percent
**Social support**		
Strong support	36	7.9
Moderate support	204	45.0
Poor support	213	47.0
**Anxiety during pregnancy**		
Yes	160	35.3
No	293	64.7
**Depression during pregnancy**		
Yes	141	31.1
No	312	68.9
**Intimate partner violence**		
Yes	110	24.3
No	343	75.7
**Perceived HRQoL**		
High	273	60.3
Low	180	39.7

### Childbirth fear and its associated factors

In this study, about 23.8% (95% CI: 20.1, 28.0) of women have experienced childbirth fear. In the multivariable logistic regression, factors such as residence, social support, intimate partner violence, and anxiety during pregnancy were significantly associated with childbirth fear.

Study participants who lived in rural areas were 6.24 times more likely to develop childbirth fear as compared to urban dwellers (AOR = 6.24, 95% CI: 3.05, 12.80). On the other hand, women who got moderate social support were 46% less likely to have childbirth fear as compared with those women who got poor social support (AOR = 0.54, 95% CI: 0.29, 0.99). The study also revealed that respondents who experienced intimate partner violence during this pregnancy were 4.95 times more likely to have childbirth fear as compared to their counterparts (AOR = 4.95, 95% CI: 2.78, 8.81). Similarly, the odds of childbirth fear among women who had anxiety during pregnancy were 2.82 times higher as compared to their counterparts (AOR = 2.82, 95% CI: 1.52, 5.23) (Table 4).

**Table 4:** Bivariable and multivariable analysis of factors associated with childbirth fear among pregnant women who were attending antenatal care in Gondar city public health facilities, northwest Ethiopia, 2022 (n = 453).

Variables	Childbirth fear		COR (95%CI)	AOR (95%CI)
	Yes	No		
**Maternal age**				
≤24 years	16	114	0.20 (0.09, 0.45)	1.03 (0.30, 3.51)
25–29 years	48	130	0.53(0.27, 1.06)	3.15 (0.96, 10.39)
30–34 years	26	75	0.50 (0.24, 1.06)	1.02 (0.33,3.10)
≥35years	18	26	1	1
**Residence**				
Rural	42	23	8.91 (5.02, 15.80)	**6.24 (3.05, 12.80)** ^**^
Urban	66	322	1	1
**Maternal educational status**				
No formal education	35	54	1.64 (0.94, 2.85)	0.79 (0.31, 0.20)
Primary education	3	45	0.17 (0.05, 0.57)	0.36 (0.10, 1.33)
Secondary education	27	137	0.50 (0.29, 0.86)	0.62 (0.32, 1.18)
College and above	43	109	1	1
**History of mental illness**				
Yes	10	11	3.10 (1.28, 7.51)	1.14 (0.32, 4.08)
No	98	334	1	1
**Gravidity**				
Primigravida	31	180	0.37 (0.23, 0.59)	0.60 (0.29, 1.21)
Multigravida	77	165	1	1
**Planned current pregnancy**				
Yes	79	308	1	1
No	29	37	3.06 (1.77, 5.27)	1.80 (0.83, 3.91)
**Social support**				
Poor social support	65	148	1	1
Moderate social support	31	173	0.41 (0.25, 0.66)	**0.54 (0.29, 0.99)** ^*^
Strong social support	12	24	1.14 (0.54, 2.41)	1.64 (0.62, 4.34)
**Anxiety during pregnancy**				
Yes	51	109	1.94 (1.25, 3.01)	**2.82 (1.52, 5.23)** ^**^
No	57	236	1	1
**Depression during pregnancy**				
Yes	47	94	2.06 (1.31, 3.22)	1.51 (0.83, 2.75)
No	61	251	1	1
**Intimate partner violence**				
Yes	58	52	6.54 (4.05, 10.56)	**4.95 (2.78, 8.81)** ^**^
No	50	293	1	

## Discussion

This institution-based cross-sectional study tried to assess the childbirth fear and associated factors among pregnant women who were attending antenatal care at Gondar city public health institutions, northwest Ethiopia, 2022. This study showed that the prevalence of childbirth fear was 23.8% (95%CI: 20.1, 28.0). This finding is in line with previous studies conducted in turkey (20.5%) [26] and Arba Minch (24.5%) Ethiopia [8].

This study was also higher than studies conducted in Malawi-20% [6], Polish-18.4% [27], and Slovakia-9.6% [28]. The possible explanation for the variation might be due to the difference in culture and characteristics of the study participants. For instance, a study in Malawi showed that 77% of study participants had high social support. However, only 7.9% of the participants get high social support in this study. It has been evidenced that women who get high social support during pregnancy have experienced a low level of childbirth fear [29]. In addition to this, a study conducted in Malawi showed only 19.7% and 15.8% of women are primiparous and illiterate in their educational level respectively, whereas in our study, nearly half (46.6%) of study participants were primiparous and 19.8% of study participants had no formal education. Evidence supports that being primiparous and lower educational level increases the experience of childbirth fear [30–32]. In the other hand, the reason for lower prevalence of childbirth fear in Polish and Slovakia might be due to the quality of antenatal care. This may address the needs of the pregnant women in preparing them for childbirth [33].

On the other hand, the result of this study is lower than the finding reported from Wollega, Ethiopia-28.9% [9]. This variation could be related to the difference in tools used to measure the degrees of fear and the different educational status of the women. The study conducted in Wollega, Ethiopia used 12-item questions with a cutoff point mean score to declare childbirth fear. However, in this study, Wijma delivery expectation/experience questionnaire version A with a cutoff point ≥85 was used. Moreover, 26.5% of study participants in Wollega have no formal education. Evidence shows that poor educational attainment increases the level of childbirth fear during pregnancy [6].

This study revealed that study participants who lived in rural areas were 6.24 times more likely to develop childbirth fear as compared to urban dwellers. Women from urban areas may have better access to media and have a better understanding and acceptance of health-related information [34]. Women who had better health-seeking behavior and service utilization could have the opportunities to communicate with health care providers about the common physiological changes during pregnancy and birth preparedness and complication readiness plan [35]. This in turn reduces the childbirth fear [36].

The finding of this study also indicates that pregnant women who received moderate social support during pregnancy were less likely to have childbirth fear than women who received poor social support during pregnancy. The odds of childbirth fear were decreased by 46.3% among pregnant women who had moderate social support compared to their counterparts. This finding is supported by the previous studies conducted in Norway [37], Turkey [25], Thailand [38], Arba Minch town, southern Ethiopia [8], and West Wollega Zone, Ethiopia [9]. The possible explanation might be social support, especially from their family, partner, neighbor, and friends are very important for the maintenance of mental health, increasing an individual’s capacity of coping with stressful situations. Moreover, pregnant women may get information, confidence, and assistance during social support so these all may decrease their childbirth fear [39]. In addition evidence showed that social support played a mediating role between depressive symptoms and childbirth fear [40]. In our study, moderate social support has a protective effect against childbirth fear, but not strong social support. This difference may be due to frequency variation between strong and moderate social support (7.9% vs 45%), variables which have high frequencies will have a high chance to significantly associate with the outcome variable.

The current study also found that intimate partner violence was a significant predicting factor for childbirth fear. Thus, Women who experienced intimate partner violence during the current pregnancy were 4.92 times more likely to experience childbirth fear than their counterparts. This finding is supported by a study conducted in Turkey and Iran [41,42]. The possible reason might be intimate partner violence during pregnancy affects a woman’s physical and mental health, pregnant women experiencing IPV reported high levels of anxiety and depression. This psychological problem and lack of support from the partner may lead the women to childbirth fear [43,44].

This study also revealed that the odds of developing childbirth fear were 2.82 times higher among women who had pregnancy-related anxiety as compared with their counterparts. This finding is supported by a previous study conducted in Turkey [45]. evidence showed that comorbidity (anxiety, depression, and childbirth fear) is very common [46].

This study collects data on the childbirth fear and related factors. The finding implies that, childbirth fear among pregnant women is high. It emphasizes the need for addressing factors related with childbirth fear with appropriate intervention is crucial to reduce childbirth fear and to improve maternal and neonatal outcome. It guides policymakers to design and implement routine screening for childbirth fear and other mental health conditions during antenatal visit to improve maternal and fetal outcome. Health care providers can also use the identified factors to develop person centered intervention to reduce childbirth fear.

### Limitation of the study

Even if female data collectors were used, this study may share the drawback of social desirability bias.

## Conclusion

In this study, the magnitude of childbirth fear is high. Rural residents, intimate partner violated women, and women who experience anxiety have increased odds of childbirth fear whereas women who get moderate support have decreased odds of childbirth fear. Therefore, policymakers should develop strategies or a screening tool for early identification of women with pregnancy-related anxiety and childbirth fear and the need to give psychological support. Healthcare providers also should pay special attention and counseling for pregnant women living in rural areas and victimized by their intimate partners. Lastly, we suggest for researchers community based study for generalizability and a longitudinal study for better detection of childbirth fear throughout pregnancy.

## Supporting information

S1 File English version questionnaire. (DOCX)

S2 File Dataset. (SAV)

## Abbreviations

ANC: Antenatal Care
AOR: Adjusted Odds Ratio
CI: Confidence Interval
COR: Crude Odds Ratio
EPDS: Edinburgh Postnatal Depression Scale
HRQoL: Health related quality of life
SPSS: Statistical Package for Social Science
WHO: World Health Organization
W-DEQ: Wijma Delivery Expectation/ Experience Questionnaire

## References

[1] Namujju J, Muhindo R, Mselle LT, Waiswa P, Nankumbi J, Muwanguzi P. Childbirth experiences and their derived meaning: A qualitative study among postnatal mothers in Mbale regional referral hospital, Uganda. Reprod Health. 2018;15(1):1–11. 30390685 10.1186/s12978-018-0628-y PMC6215682

[2] Okumus F, Sahin N. Fear of childbirth in urban and rural regions of Turkey: Comparison of two resident populations. North Clin Istanb. 2017;4(3):247–56. doi: 10.14744/nci.2017.46693 29270574 PMC5724920

[3] Nilsson C, Hessman E, Sjöblom H, Dencker A, Jangsten E, Mollberg M. Definitions, measurements and prevalence of fear of childbirth: A systematic review. BMC Pregnancy Childbirth. 2018;18(1):1–15. 29329526 10.1186/s12884-018-1659-7 PMC5766978

[4] O’Connell MA, Khashan AS, Leahy-Warren P, Stewart F, O’Neill SM. Interventions for fear of childbirth including tocophobia. Cochrane Database Syst Rev. 2021;7(7):CD013321. doi: 10.1002/14651858.CD013321.pub2 34231203 PMC8261458

[5] Sanjari S, Chaman R, Salehin S, Goli S, Keramat A. Update on the global prevalence of severe fear of childbirth in low-risk pregnant women: A systematic review and meta-analysis. Int J Women’s Heal Reprod Sci. 2022;10(1):3–10.

[6] Khwepeya M, Lee GT, Chen S-R, Kuo S-Y. Childbirth fear and related factors among pregnant and postpartum women in Malawi. BMC Pregnancy Childbirth. 2018;18(1):391. doi: 10.1186/s12884-018-2023-7 30285754 PMC6171200

[7] Onchonga D, Moghaddam Hosseini V, Keraka M, Várnagy Á. Prevalence of fear of childbirth in a sample of gravida women in Kenya. Sex Reprod Healthc. 2020;24. 10.1016/j.srhc.2020.100510 32217359

[8] Gelaw T, Ketema TG, Beyene K, Gurara MK, Ukke GG. Fear of childbirth among pregnant women attending antenatal care in Arba Minch town, southern Ethiopia: a cross-sectional study. BMC Pregnancy Childbirth. 2020;20(1):672. doi: 10.1186/s12884-020-03367-z 33160330 PMC7648954

[9] Berhanu RD, Abathun AD, Negessa EH, Amosa LG. The magnitude and associated factors of childbirth fear among pregnant women attending antenatal care at public hospitals in Ethiopia: a cross-sectional study. BMC Pregnancy Childbirth. 2022;22(1):222. doi: 10.1186/s12884-022-04544-y 35305600 PMC8933614

[10] Dencker A, Nilsson C, Begley C, Jangsten E, Mollberg M, Patel H, et al. Causes and outcomes in studies of fear of childbirth: A systematic review. Women Birth. 2019;32(2):99–111. doi: 10.1016/j.wombi.2018.07.004 30115515

[11] Sydsjö G, Angerbjörn L, Palmquist S, Bladh M, Sydsjö A, Josefsson A. Secondary fear of childbirth prolongs the time to subsequent delivery. Acta Obstet Gynecol Scand. 2013;92(2):210–4. doi: 10.1111/aogs.12034 23066797

[12] Klabbers GA, Wijma K, Paarlberg KM, Emons WHM, Vingerhoets AJJM. Haptotherapy as a new intervention for treating fear of childbirth: a randomized controlled trial. J Psychosom Obstet Gynaecol. 2019;40(1):38–47. doi: 10.1080/0167482X.2017.1398230 29157055

[13] Mortazavi F, Agah J. Childbirth Fear and Associated Factors in a Sample of Pregnant Iranian Women. Oman Med J. 2018;33(6):497–505. doi: 10.5001/omj.2018.91 30410692 PMC6206413

[14] Onchonga D. Prenatal fear of childbirth among pregnant women and their spouses in Kenya. Sex Reprod Healthc. 2021;27:100593. doi: 10.1016/j.srhc.2020.100593 33421700

[15] Nations U. The 2030 Agenda and the Sustainable Development Goals: An Opportunity for Latin America and the Caribbean. Santiago: United Nations. 2018.

[16] Health M of. Health Sector Transformation Plan II (HSTP II) 2020/21 - 2024/25 (2013 EFY-2017 EFY). 2021.

[17] Central Statistical Agency Et. Population Projection of Ethiopia for All Regions at Wereda Level from 2014 – 2017. 2013.

[18] Wijma K, Wijma B, Zar M. Psychometric aspects of the W-DEQ; a new questionnaire for the measurement of fear of childbirth. J Psychosom Obstet Gynaecol. 1998;19(2):84–97. doi: 10.3109/01674829809048501 9638601

[19] Beyene GM, Azale T, Gelaye KA, Ayele TA. The effect of antenatal depression on birth weight among newborns in South Gondar zone, Northwest Ethiopia: a population-based prospective cohort study. Arch Public Health. 2021;79(1):121. doi: 10.1186/s13690-021-00643-y 34225799 PMC8256480

[20] Wall V, Premji SS, Letourneau N, McCaffrey G, Nyanza EC. Factors associated with pregnancy-related anxiety in Tanzanian women: a cross sectional study. BMJ Open. 2018;8(6):e020056. doi: 10.1136/bmjopen-2017-020056 29866722 PMC5988139

[21] Abiola T, Udofia O, Psychiatry MZMJ of. Psychometric properties of the 3-item oslo social support scale among clinical students of Bayero University Kano, Nigeria. M Z M J Psychiatry. 2013;22(2):1–10.

[22] Azene ZN, Yeshita HY, Mekonnen FA. Intimate partner violence and associated factors among pregnant women attending antenatal care service in Debre Markos town health facilities, Northwest Ethiopia. PLoS One. 2019;14(7):e0218722. doi: 10.1371/journal.pone.0218722 31260469 PMC6602189

[23] Tola Y, Ayele G, Boti N, Yihune M, Gethahun F, Gebru Z. Health-Related Quality-of-Life and Associated Factors Among Post-Partum Women in Arba Minch Town. Int J Womens Health. 2021;13:601–11. doi: 10.2147/IJWH.S295325 34188554 PMC8232860

[24] Asresu TT, Hailu D, Girmay B, Abrha MW, Weldearegay HG. Mothers’ utilization and associated factors in preconception care in northern Ethiopia: A community based cross sectional study. BMC Pregnancy Childbirth. 2019;19(1):1–7. 31601190 10.1186/s12884-019-2478-1 PMC6787988

[25] İsbir G, Serçekuş P, Yenal K, Okumuş H, Durgun Ozan Y, Karabulut Ö. The prevalence and associated factors of fear of childbirth among Turkish pregnant women. J Reprod Infant Psychol. 2022. 10.1080/02646838.2022.2057938 35345941

[26] Kaya D, Evcili F. The affecting factors of childbirth fear for pregnant women admitted to a health center and university hospital in Turkey. J Heal Res. 2020;34(5):389–97.

[27] Michalina I, Anna BS, Anna KZ, Ewa B, Hanna G, Wojciech C. Factors associated with fear of childbirth among Polish pregnant women. Sci Rep. 2021;11(1):1–8. 33623084 10.1038/s41598-021-83915-5 PMC7902668

[28] Mazúchova L, Skodova Z, Kelčíkova S, Rabárova A. Factors associated with childbirth – related fear among Slovak women. Cent Eur J Nurs Midwifery. 2017;8(4):742–8.

[29] Zhou X, Liu H, Li X, Zhang S. Fear of childbirth and associated risk factors in healthy pregnant women in northwest of china: a cross-sectional study. Psychol Res Behav Manag. 2021;14:731–41. doi: 10.2147/PRBM.S309889 34135648 PMC8200453

[30] Soysal C, Işıkalan MM. Determination of risk factors affecting fear of childbirth during pregnancy. Cukurova Med J. 2020;45(4):1340–5.

[31] Shakarami A, Mirghafourvand M, Abdolalipour S, Jafarabadi MA, Iravani M. Comparison of fear, anxiety and self-efficacy of childbirth among primiparous and multiparous women. BMC Pregnancy Childbirth. 2021;21(1):642. doi: 10.1186/s12884-021-04114-8 34548055 PMC8456545

[32] Green G, Tesler R, Marques A. Primiparous and Multiparous Women’s Mode of Birth and Negative Emotions. Int J Environ Res Public Health. 2022;19(9):5189. doi: 10.3390/ijerph19095189 35564584 PMC9103235

[33] Alizadeh-Dibazari Z, Abdolalipour S, Mirghafourvand M. The effect of prenatal education on fear of childbirth, pain intensity during labour and childbirth experience: a scoping review using systematic approach and meta-analysis. BMC Pregnancy Childbirth. 2023;23(1):541. doi: 10.1186/s12884-023-05867-0 37501120 PMC10373291

[34] Nuamah GB, Agyei-Baffour P, Mensah KA, Boateng D, Quansah DY, Dobin D, et al. Access and utilization of maternal healthcare in a rural district in the forest belt of Ghana. BMC Pregnancy Childbirth. 2019;19(1):6. doi: 10.1186/s12884-018-2159-5 30612557 PMC6322319

[35] Musa A, Amano A. Determinants of birth preparedness and complication readiness among pregnant woman attending antenatal care at Dilchora Referral Hospital, Dire Dawa City, East Ethiopia. Gynecol Obstet. 2016;6(2).

[36] Kempe A, Theorell T, Alwazer FNA, Taher SA, Christensson K. Exploring women’s fear of childbirth in a high maternal mortality setting on the Arabian Peninsula. Glob Ment Heal (Cambridge, England). 2015;2. 10.1017/gmh.2015.6 PMC5269624 28596858

[37] Størksen HT, Garthus-Niegel S, Adams SS, Vangen S, Eberhard-Gran M. Fear of childbirth and elective caesarean section: a population-based study. BMC Pregnancy Childbirth. 2015;15:221. doi: 10.1186/s12884-015-0655-4 26382746 PMC4573308

[38] Phunyammalee M, Buayaem T, Boriboonhirunsarn D. Fear of childbirth and associated factors among low-risk pregnant women. J Obstet Gynaecol. 2019;39(6):763–7. 31007101 10.1080/01443615.2019.1584885

[39] Azimi M, Fahami F, Mohamadirizi S. The Relationship between Perceived Social Support in the First Pregnancy and Fear of Childbirth. Iran J Nurs Midwifery Res. 2018;23(3):235–9. doi: 10.4103/ijnmr.IJNMR_170_16 29861764 PMC5954647

[40] Zhou X-L, Liu H, Li X-H, Li F, Zhang S-M, Zhang S-R. Mediating effects of social support between antenatal depression and fear of childbirth among nulliparous woman. Ann Palliat Med. 2021;10(6):6399–409. doi: 10.21037/apm-21-854 34237961

[41] Oğurlu M, Erbil N. The Effect of Intimate Partner Violence on Fear of Childbirth Among Pregnant Women. J Interpers Violence. 2023;38(3–4):3737–55. doi: 10.1177/08862605221109915 35876023

[42] Moghaddam Hossieni V, Toohill J, Akaberi A, HashemiAsl BM. Influence of intimate partner violence during pregnancy on fear of childbirth. Sex Reprod Healthc. 2017;14:17–23. 29195630 10.1016/j.srhc.2017.09.001

[43] Toohill J, Fenwick J, Gamble J, Creedy DK, Buist A, Ryding EL. Psycho-Social Predictors of Childbirth Fear in Pregnant Women: An Australian Study. OJOG. 2014;04(09):531–43. doi: 10.4236/ojog.2014.49075

[44] Howard LM, Oram S, Galley H, Trevillion K, Feder G. Domestic violence and perinatal mental disorders: a systematic review and meta-analysis. PLoS Med. 2013;10(5):e1001452. doi: 10.1371/journal.pmed.1001452 23723741 PMC3665851

[45] Yılmaz FA, Karakurt P, C NY. The Relationship between the Fear of Childbirth and Anxiety during the Covid-19 Pandemic. 2022;29(2).

[46] Hildingsson I, Nilsson J, Merio E, Larsson B. Anxiety and depressive symptoms in women with fear of birth: A longitudinal cohort study. 2021;:1–9. 10.18332/ejm/138941 PMC8328228 34396062

