# Supplementary material for: Childbirth fear and its associated factors among pregnant women attending antenatal care at Gondar city public health institutions, northwest Ethiopia, 2022
Source: PLoS One. 2025 Jul 23;20(7):e0328819. doi: 10.1371/journal.pone.0328819 (PMC12286399; doi:10.1371/journal.pone.0328819)
Supplement: S1 File — (DOCX) [file pone.0328819.s001.docx]

**English version questionnaire**

**Part I: sociodemographic characteristics of respondents**

| S.N | Question | Answers | skip |
| --- | --- | --- | --- |
| 1 | What is your age in complete years? | ----------------years |  |
| 2 | Where is your place of residence? | 1. Rural 2. Urban |  |
| 3 | What is your religion? | 1. Orthodox 2. Muslim 3. Protestant 4. Catholic 5. Others (Specify)_____ |  |
| 4 | What is your educational status? | Unable to read and write  Able to read and write   1. Primary education 2. Secondary education 3. College and above |  |
| 5 | What is your occupation? | 1. Government employed 2. Housewife 3. Self employed 4. Student 5. Other specify---------------- |  |
| 6 | What is your current marital status? | 1. Marred 2. Single 3. Divorced 4. Widowed |  |
| 7 | What is your partner’s educational status? | Unable to read and write  Able to read and write   1. Primary education 2. Secondary education 3. College and above |  |
| 8 | What is your partner’s occupation? | 1. Farmer 2. Student 3. Private employee 4. Government employee 5. Merchant   Others (specify)_______ |  |

**Part II: Obstetrics/reproductive related questions**

| 1 | What is your age at first marriage? | _____________Years. |  |
| --- | --- | --- | --- |
| 2 | How many times did you get pregnant? |  |  |
| 3 | What is your Gestational age in complete weeks( estimated based on LNMP or from early Ultrasound) | --------------------weeks |  |
| 4 | Is your currenet pregnancy planned? | 1. Yes 2. No |  |
| 5 | Is the current pregnancy wanted? | 1. Yes 2. No |  |
| 6 | Have you received preconception care in the current pregnancy? | 1. Yes 2. No |  |
| 7 | What was the mode of delivery of last baby? | 1. Vaginal birth 2. Cesarean section 3. Instrumental delivery |  |
| 8 | What is your Preference for mode of delivery | 1. Vaginal birth 2. Cesarean section 3. Instrumental delivery |  |
| 9 | Do you experience Complication during past pregnancy | 1. Yes 2. No |  |
| 10 | If yes for question no 9 which complication | 1. Pregnancy induced hypertension 2. Gestational diabetes mellitus 3. Anemia 4. Vaginal bleeding 5. Complications during labour & delivery 6. Other (specify)………….. |  |
| 11 | Have you ever experienced adverse pregnancy outcomes? | 1. Yes 2. No |  |
| 12 | Which type of adverse pregnancy outcome have you experienced? (multiple responses are possible) | 1. Congenital anomalies 2. Low birth weight 3. Preterm 4. Abortion 5. Still birth 6. Neonatal death 7. Others (specify)……....... |  |
| 13 | Do you have history chronic medical illness | 1. Yes 2. No |  |
| 14 | Have you diagnosed mental illness by health care provider currently or in the past life? | 1. Yes 2. No |  |

**Part III: The Wijma Delivery Expectancy Experience Questionnaire (W-DEQ) version A**

| I | How do you think your labour and delivery will turn  out **as** a whole? | 1/ extremely | 2 | 3 | 4 | 5 | 6/not at all |
| --- | --- | --- | --- | --- | --- | --- | --- |
| 1 | **Fantastic** | Extremely  fantastic |  |  |  |  | Not at all  fantastic |
| 2 | **Frightful** | Extremely  frightful |  |  |  |  | Not at all  frightful |
| II | How do **you** think **you** will feel in general during the  labour and delivery! |  |  |  |  |  |  |
| 3 | **Lonely** | Extremely  lonely |  |  |  |  | Not at all  lonely |
| 4 | **Strong** | Extremely  strong |  |  |  |  | Not at all  strong |
| 5 | **Confident** | Extremely  confident |  |  |  |  | Not at all  confident |
| 6 | **Afraid** | Extremely  afraid |  |  |  |  | Not at all  afraid |
| 7 | **Deserted** | Extremely  deserted |  |  |  |  | Not at all  deserted |
| 8 | **Weak** | Extremely  weak |  |  |  |  | Not at all  weak |
| 9 | **Safe** | Extremely  safe |  |  |  |  | Not at all  safe |
| 10 | **Independent** | Extremely  independent |  |  |  |  | Not at all  independent |
| 11 | **Desolate** | Extremely  desolate |  |  |  |  | Not at all  desolate |
| 12 | **Tense** | Extremely  tense |  |  |  |  | Not at all  tense |
| 13 | **Glad** | Extremely  glad |  |  |  |  | Not at all  glad |
| 14 | **Proud** | Extremely  proud |  |  |  |  | Not at all  proud |
| 15 | **Abandoned** | Extremely  abandoned |  |  |  |  | Not at all  abandoned |
| 16 | **Composed** | Totally  composed |  |  |  |  | Not at all  composed |
| 17 | **Relaxed** | Extremely  relaxed |  |  |  |  | Not at all  relaxed |
| 18 | **Happy** | Extremely happy |  |  |  |  | Not at all happy |
| III | How do you think you will feel during the labour and delivery? |  |  |  |  |  |  |
| 19 | **Panic** | Extreme  panic |  |  |  |  | No panic  at all |
| 20 | **Hopelessness** | Extreme hopelessness |  |  |  |  | No hopelessness |
| 21 | **Longing for the child** | Extreme longing for  the child |  |  |  |  | No longing  for the child  at all |
| 22 | **Self-confidence** | Extreme self-  confidence |  |  |  |  | No self-  confidence  at all |
| 23 | **Trust** | Extreme trust |  |  |  |  | No trust at al |
| 24 | **Pain** | Extreme pain |  |  |  |  | No pain at all |
| IV | What do you think will happen when labour is most intense? |  |  |  |  |  |  |
| 25 | **Behave badly** | I will behave  extremely  badly |  |  |  |  | I will not behave  badly at all |
| 26 | **Let happen** | I will dare to  surrender  control to  my body |  |  |  |  | I will not  totally dare to  surrender control to my body  at all |
| 27 | **Lose control** | I will totally  lose control  of myself |  |  |  |  | I will not  lose control of myself  at all |
| V | How do you imagine it will feel the very moment?  you deliver the baby? |  |  |  |  |  |  |
| 28 | **Funny** | Extremely  funny |  |  |  |  | Not at all funny |
| 29 | **Natural** | Extremely  natural |  |  |  |  | Not at all  natural |
| 30 | **Obvious** | Extremely  self-evident |  |  |  |  | Not at all  self-evident |
| 31 | **Dangerous** | Extremely  dangerous |  |  |  |  | Not at all  dangerous |
| VI | Have you, during the last month. had fantasies  about the labour and delivery, for example. |  |  |  |  |  |  |
| 32 | **Child will die** | Never |  |  |  |  | Very often |
| 33 | **Child will** be **injured** | Never |  |  |  |  | Very often |

**Part IV: quality of life**

|  | **Physical domain** | 1/not at all | 2/a little | 3/amoderate amount | 4/very much | 5/extreme amount |
| --- | --- | --- | --- | --- | --- | --- |
| 1 | How well are you able to get around |  |  |  |  |  |
| 2 | To what extent do you feel that (physical) pain prevents you from doing what you need to do? |  |  |  |  |  |
| 3 | Do you have enough energy for everyday life? |  |  |  |  |  |
| 4 | How satisfied are you with your sleep? |  |  |  |  |  |
| 5 | How satisfied are you with your ability to perform your daily living activities? |  |  |  |  |  |
| 6 | How much do you need any medical treatment to function in your daily life |  |  |  |  |  |
| 7 | How satisfied are you with your capacity for work? |  |  |  |  |  |
|  | **Psychological domain** |  |  |  |  |  |
| 8 | How much do you enjoy life |  |  |  |  |  |
| 9 | How well are you able to concentrate? |  |  |  |  |  |
| 10 | How satisfied are you with yourself? |  |  |  |  |  |
| 11 | Are you able to accept your bodily appearance? |  |  |  |  |  |
| 12 | To what extent do you feel your life to be meaningful? |  |  |  |  |  |
| 13 | How often do you have negative feelings, such as blue mood, despair, anxiety,depression? |  |  |  |  |  |
|  | **Social relationships** |  |  |  |  |  |
| 14 | How satisfied are you with your sex life? |  |  |  |  |  |
| 15 | How satisfied are you with your personal relationship? |  |  |  |  |  |
| 16 | How satisfied are you with the support you get from your friend? |  |  |  |  |  |
|  | **Environmental domain** |  |  |  |  |  |
| 17 | How satisfied are you with the conditions of your living place? |  |  |  |  |  |
| 18 | Have you enough money to meet your needs? |  |  |  |  |  |
| 19 | How satisfied are you with your access to health service? |  |  |  |  |  |
| 20 | How healthy is you’re your physical environment? |  |  |  |  |  |
| 21 | To what extent do you have the opportunity for leisure activities? |  |  |  |  |  |
| 22 | How safe do you feel in your daily life? |  |  |  |  |  |
| 23 | How satisfied are you with your transport? |  |  |  |  |  |
| 24 | How available to you is the information that you need in your day-to-day life? |  |  |  |  |  |
| 25 | How would you rate your quality of life? | 1.Very poor | 2. poor | 3.Neither poor nor good | 4.good | 5.very good |
| 26 | How satisfied are you with your health? | 1.Very poor | 2. poor | 3.Neither poor nor good | 4.good | 5.very good |

**Part V: Oslo-3 items social support scale**

| 1 | How easy can you get help from neighbors | 1, Very difficult 2, Difficult 3, Possible  4, Easy 5, Very easy |  |
| --- | --- | --- | --- |
| 2 | How many people are so close to you that you can count on them if you have serious problems? | -----------------persons |  |
| 3 | How much concern do people show in what you are doing? | 1, No 2, Little 3, Uncertain  4, Some 5, A lot |  |

**Part VI: Edinburgh Perinatal/Postnatal Depression Scale (EPDS)**

Instruction: Tell us the way you have been feeling in the past 7 days including today

| 1 | In the past 7 days, have you been able to laugh and see the funny side of things | 1. 0 As much as I always could 2. 1 Not quite so much now 3. 2 Definitely not so much now   3 Not at all |  |
| --- | --- | --- | --- |
| 2 | In the past 7 days, have you looked forward with enjoyment to things | 0 As much as I ever did  1 Rather less than I used to  2 Definitely less than I used to  3 Hardly at all |  |
| 3 | In the past 7 days, have you blamed yourself unnecessarily when things went wrong | 3 Yes, most of the time  2 Yes, some of the time   1. 1 Not very often   0 No, never |  |
| 4 | In the past 7 days, have you been anxious or worried for no good reason | 1. 0 No, not at all   1 Hardly ever  2 Yes, sometimes  3 Yes, very often |  |
| 5 | In the past 7 days, have you felt scared or panicky for no very good reason | 3 Yes, quite a lot  2 Yes, sometimes  1 No, not much  0 No, not at all |  |
| 6 | In the past 7 days, have things been getting on top of you | 3 Yes, most of the time I haven’t been able to cope  2 Yes, sometimes I haven’t been coping as well as usual  1 No, most of the time I have coped quite well  0 No, I have been coping as well as ever |  |
| 7 | In the past 7 days, have you been so unhappy that you have had difficulty  sleeping | 3 Yes, most of the time  2 Yes, sometimes  1 Not very often  0 No, not at all |  |
| 8 | In the past 7 days, have you felt sad or miserable | 3 Yes, most of the time  2 Yes, quite often  1 Not very often  0 No, not at all |  |
| 9 | In the past 7 days, have you been so unhappy that you have been crying | 3 Yes, most of the time  2 Yes, most of the time  1 Only occasionally  0 No, never |  |
| 10 | In the past 7 days, the thought of harming yourself has occurred to you | 3 Yes, quite often  2 Sometimes  1 Hardly ever  0 Never |  |

**Part VII: Pregnancy-related anxiety Questionnaire (PRA-Q)**

|  | | 0 | 1 | 2 | 3 |
| --- | --- | --- | --- | --- | --- |
| 1 | I am worried about the delivery. |  |  |  |  |
| 2 | I am worried about the pain of contractions and the pain during delivery. |  |  |  |  |
| 3 | I am worried about not being able to control myself during labor and fear that I will scream. |  |  |  |  |
| 4 | I am worried about the fact that I shall not regain my figure after delivery. |  |  |  |  |
| 5 | I am concerned about my unattractive appearance. |  |  |  |  |
| 6 | I am worried about my enormous weight gain. |  |  |  |  |
| 7 | I think that our child will be in poor health or will be prone to illnesses. |  |  |  |  |
| 8 | I am afraid the baby will be mentally handicapped or will suffer from brain damage. |  |  |  |  |
| 9 | I am afraid our baby will be stillborn, or will die during or immediately after delivery. |  |  |  |  |
| 10 | I am afraid that our baby will suffer from a physical defect or worry that something will be physically. |  |  |  |  |

**Note:** 0= Never, 1=hardly ever, 2=sometimes, 3= Yes, quite often

**Part VIII: Intimate partner violence related questions**

|  | **Psychological violence** |  |  |
| --- | --- | --- | --- |
| 1 | Did your partner insulted you or made you feel bad about yourself | 1. Yes 2. No |  |
| 2 | Did your partner belittled or humiliated you infront of others | 1. Yes 2. No |  |
| 3 | Did your partner scared or intimidated on purpose | 1. Yes 2. No |  |
| 4 | Did your partner threatened when visiting friends/family | 1. Yes 2. No |  |
|  | **Physical violence** |  |  |
| 5 | Did your partner slapped you or thrown something at you that could hurt you | 1. Yes 2. No |  |
| 6 | Did your partner pushed you or shoved or pulled your hair | 1. Yes 2. No |  |
| 7 | Did your partner hit you with his fist or with something else that could hurt you | 1. Yes 2. No |  |
| 8 | Did your partner beaten in the abdomen | 1. Yes 2. No |  |
| 9 | Did your partner choked or burnt you on purpose | 1. Yes 2. No |  |
| 10 | Did your partner threatened to use or actually used a gun, knife, or any other weapon against you | 1. Yes 2. No |  |
|  | **Sexual violence** |  |  |
| 11 | Did your partner physically forced you to have sexual intercourse | 1. Yes 2. No |  |
| 12 | Did you have an experience of unwanted sexual intercourse because of fear from the partner | 1. Yes 2. No |  |
| 13 | Did your partner forced you to do something sexual that is degrading or humiliating | 1. Yes 2. No |  |

**Thank you!!!**
